# Feasibility Study on Applying Radiophotoluminescent Glass Dosimeters for CyberKnife SRS Dose Verification

**DOI:** 10.1371/journal.pone.0169252

**Published:** 2017-01-03

**Authors:** Shih-Ming Hsu, Chao-Hsiung Hung, Yi-Jen Liao, Hsiao-Mei Fu, Jo-Ting Tsai, Yung-Hui Huang, David Y. C. Huang

**Affiliations:** 1 Medical Physics and Radiation Measurements Laboratory, Department of Biomedical Imaging and Radiological Sciences, National Yang-Ming University, Taipei, Taiwan, ROC; 2 Department of Biomedical Imaging and Radiological Sciences, National Yang-Ming University, Taipei, Taiwan, ROC; 3 Biophotonics and Molecular Imaging Research Center, National Yang-Ming University, Taipei, Taiwan, ROC; 4 Division of Radiation Therapy and Oncology, Chang Gung Memorial Hospital, Chia-Yi, Taiwan, ROC; 5 School of Medical Laboratory Science and Biotechnology, Collage of Medical Science and Technology, Taipei Medical University, Taipei, Taiwan, ROC; 6 Department of radiation Oncology, MacKay Memorial Hospital, New-Taipei, Taiwan, ROC; 7 CyberKnife Treatment Center, Taipei Medical University-Wan Fang Hospital, Taipei, Taiwan, ROC; 8 Department of Medical Imaging and Radiological Sciences, I-Shou University, Taiwan, R.O.C.; 9 Medical Physics Graduate Program at Duke Kunshan University, Kunshan, Jiangsu, China; North Shore Long Island Jewish Health System, UNITED STATES

## Abstract

CyberKnife is one of multiple modalities for stereotactic radiosurgery (SRS). Due to the nature of CyberKnife and the characteristics of SRS, dose evaluation of the CyberKnife procedure is critical. A radiophotoluminescent glass dosimeter was used to verify the dose accuracy for the CyberKnife procedure and validate a viable dose verification system for CyberKnife treatment. A radiophotoluminescent glass dosimeter, thermoluminescent dosimeter, and Kodak EDR2 film were used to measure the lateral dose profile and percent depth dose of CyberKnife. A Monte Carlo simulation for dose verification was performed using BEAMnrc to verify the measured results. This study also used a radiophotoluminescent glass dosimeter coupled with an anthropomorphic phantom to evaluate the accuracy of the dose given by CyberKnife. Measurements from the radiophotoluminescent glass dosimeter were compared with the results of a thermoluminescent dosimeter and EDR2 film, and the differences found were less than 5%. The radiophotoluminescent glass dosimeter has some advantages in terms of dose measurements over CyberKnife, such as repeatability, stability, and small effective size. These advantages make radiophotoluminescent glass dosimeters a potential candidate dosimeter for the CyberKnife procedure. This study concludes that radiophotoluminescent glass dosimeters are a promising and reliable dosimeter for CyberKnife dose verification with clinically acceptable accuracy within 5%.

## Introduction

The rapid development of computer technology in recent years has brought new radiation therapy techniques to replace traditional stereotactic radiosurgery (SRS) methods. Advances in SRS have led to better dissection of microlesions that are difficult to surgically remove and have improved the quality of life for patients. SRS consists of small field radiation therapy and is commonly achieved by one of the following systems: Gamma knife, X knife (cone or intensity-modulated technique (IMRT)), or CyberKnife.

SRS delivers high dose radiation and is typically completed in one to three fractions, whereas conventional radiation therapy is completed in 20 to 30 fractions. However, for the dose verification of small field radiation therapy techniques, the influence of lateral electron disequilibrium and high dose gradients could increase the inaccuracy of dose measurements [1–4]. CyberKnife has various small circular collimator sizes; therefore, dose evaluation is critical. Monte Carlo-based dose calculations have become major tools for the verification of clinical dosimetry in small field radiation therapy techniques [5–10]. Ling [11] reported that a pencil beam algorithm can be used in Cyberknife system. Ling developed a model based dose calculation algorithm to better handle the lateral scatter in an irregularly shaped small field for the CyberKnife system.

This study uses the Monte Carlo BEAMnrc to simulate the percent depth dose and lateral dose profile of Accuray G3 CyberKnife (Accuray Inc, USA). The simulated results are compared to the actual measurements. In this study, thermoluminescent dosimeters (TLDs), radiophotoluminescent glass dosimeters (RPLGDs), and Kodak EDR2 films were used to measure the lateral dose profile and percent depth dose of CyberKnife. Araki [6] reported that a large active volume, high density, and high atomic number dosimeters affected the measured results for CyberKnife. Therefore, an anthropomorphic phantom was also used in this study to evaluate the accuracy of the dose supplied by CyberKnife and assess the feasibility of the clinical dose verification using RPLGD, TLD, and Kodak EDR2 film.

## Materials and Methods

### CyberKnife

Early models of CyberKnife (Accuray Inc., USA) were mainly used to treat intracranial lesions. The newer model has broader applications for various tumor sites [12–14]. The features of G3 CyberKnife include a 6 MV X-band Linac on a mechanical arm that is capable of creating more than 1200 treatment beams in 3D space with six axes and narrow beams collimated by 12 secondary collimators with different circular collimator sizes and two sets of image devices for image-guided radiotherapy (IGRT). CyberKnife adopted a non-isocentric treatment method to allow the dose profiles to conform to the distribution of the tumor shape and minimize the damage to the normal tissue surrounding the tumor.

### Monte Carlo simulations

**Fig 1:**
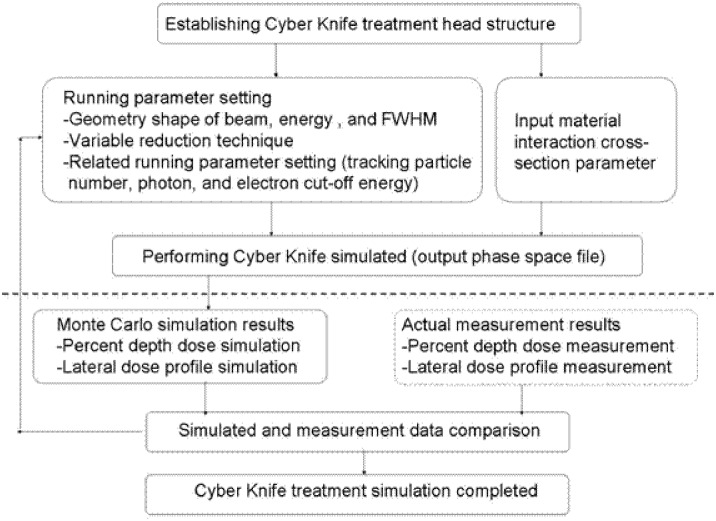
Flowchart of the Monte Carlo program simulation.

The Monte Carlo (MC) simulation code (OMEGA/BEAM) used in the study was developed by the National Research Council of Canada (NRCC) and the University of Wisconsin. A flowchart of the OMEGA/BEAM simulation is shown in Fig 1. Bremsstrahlung splitting and Russian roulette variable reduction techniques were used to increase the relative particle collection efficiency. The photon cutoff energy was set at 0.01 MeV, and the electron cutoff energy was set at 0.7 MeV. The voxel size for the simulation was 1 mm^3^. In this study, we used water and acrylic as the media for the MC simulations. The results were then compared to the measured results in terms of the lateral dose profile and percent depth dose.

### Dosimeters and reader systems

#### TLD and REXON UL-320 readout system

The TLD used in this study was the Harshaw TLD-100H (LiF: Mg, Cu, P). The TLD-100H measures 1 mm^3^ in size and has an effective atomic number of 8.2 and a density of 2.64 gm/cm^3^. The TLD-100H readout system was the REXON UL-320 reader (REXON Inc., USA).

#### RPLGD and DOSE ACE FGD-1000 readout system

The RPLGD and the readout system for the dose measurement were the GD-302M glass dosimeter and Dose Ace FDG-1000 system (Asahi Techno Glass Corporation, Japan), respectively. The composition of the GD-302M is as follows: O (51.61%), P (31.55%), Na (11.00%), Al (6.12%), and Ag (0.17%) [13]. The effective atomic number of the GD-302M is 12.04, and the density is 2.61 gm/cm^3^. The glass dosimeter has a cylindrical shape and is 1.5 mm in diameter and 12 mm in length. The readout system used a 1 mm diameter pulsed UV laser as an excitation light source, and the visible light signal was then collected through the 0.6 mm reading window to evaluate the radiation dose [15].

#### Kodak EDR2 film and Lumisys LS75 readout system

The third method for evaluating the dose response was Kodak EDR2 film (Kodak, USA). The optimal dose response range for EDR2 film is between 25 to 400 cGy; saturation occurs at 700 cGy. The optical density readout system for the EDR2 film was the Lumisys LS75 laser scanner (Kodak, USA), which has a maximum resolution of 0.1 mm. EDR2 film and the PTW-Freiburg analytic MEPHYSTO software Version 7.33 were used to scan the images and determine the optical density that corresponded to the dose response.

### Characteristic analysis for the dosimeters

Radiation detection characteristic analyses of the dosimeters, including reproducibility and linearity, were also conducted to ensure accuracy.

#### Reproducibility of the dosimeters

The dosimeters were irradiated with a single dose (200 cGy) by the AECL Co-60 unit. All dosimeters were irradiated, read, and annealed 10 times to obtain the coefficient of variation (CV) to determine reproducibility. A low CV indicates better stability for the dosimeter readout and thus better reproducibility.

#### Dose linearity

Dose linearity characterizes the relative linearity between the readout and dose delivered to the dosimeters at different doses. This study used the AECL Co-60 as a source, with 25, 50, 75, 100, 125, 200, 225, 300, 325, and 400 cGy for the analysis of dose linearity.

### Lateral dose profile measurement

This study also used the TLD-100H, GD-302M, and EDR2 film for the measurement of the lateral dose profile. The lateral dose profile was measured at a depth of 5 cm in an acrylic phantom with the SSD set to 75 cm. The measured results were normalized to the dose value at the isocenter. The measurements of the lateral dose profile with the TLD-100H, GD-302M, and EDR2 film were conducted using an acrylic phantom five times for each circular collimator size (5, 10, 20, 30, 40, and 60 mm). The mean dose and CV were normalized to the isocenter dose value to obtain the lateral dose profile for each circular collimator size.

### Percent depth dose measurement

The percent depth doses were measured using two CyberKnife circular collimator sizes: 40 and 60 mm. The percent depth dose measurements with the GD-302M dosimeters were measured at an SSD of 75 cm and depths of 3, 6, 9, 12, 15, 18, 30, 60, 120, 180, and 240 mm in an acrylic phantom. Each measured depth was repeated five times to obtain the mean dose and CV. The mean dose was normalized to the depth of the maximum dose at 1.5 cm (for the 6 MV beam).

### Anthropomorphic phantom dose measurement

**Fig 2:**
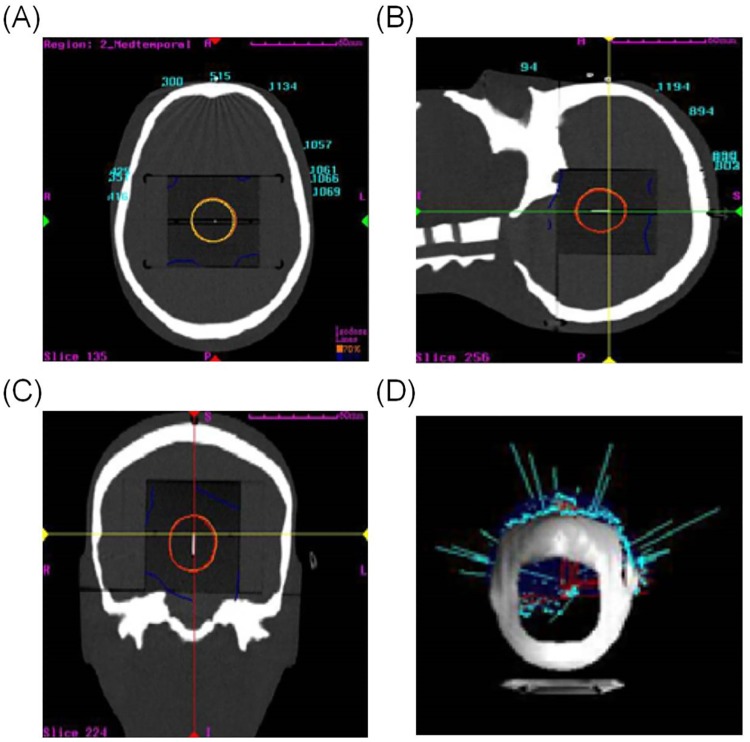
Treatment planning for an anthropomorphic phantom. (A) and (B) are the axial and sagittal plane images. (C) is the coronal plane image. The circular area is the simulated target. (D) is the schematic diagram for incident beam directions.

The GD-302M dosimeter was used to evaluate the accuracy of the dose given by the CyberKnife to an anthropomorphic phantom (Accuray Inc., USA). This study used polystyrene phantoms of 63.5 mm in both length and width and 3 mm in thickness to replace the radiochromic film pack in the anthropomorphic phantom. The GD-302M dosimeter was placed in the polystyrene phantoms for the dose measurement. For the simulated target shown in Fig 2, the target was a circular shape with a diameter of 3 cm. The CyberKnife treatment planning system used 6 dimensional cranial tracking modules to deliver the treatment dose. The dose given to the target was 3000 cGy in 3 fractions (1000 cGy per fraction). During the irradiation process, the target localization system was used to track the tumor location to ensure the accuracy of delivery.

## Results and Discussion

### Radiation detection characteristics of the dosimeters

**Fig 3:**
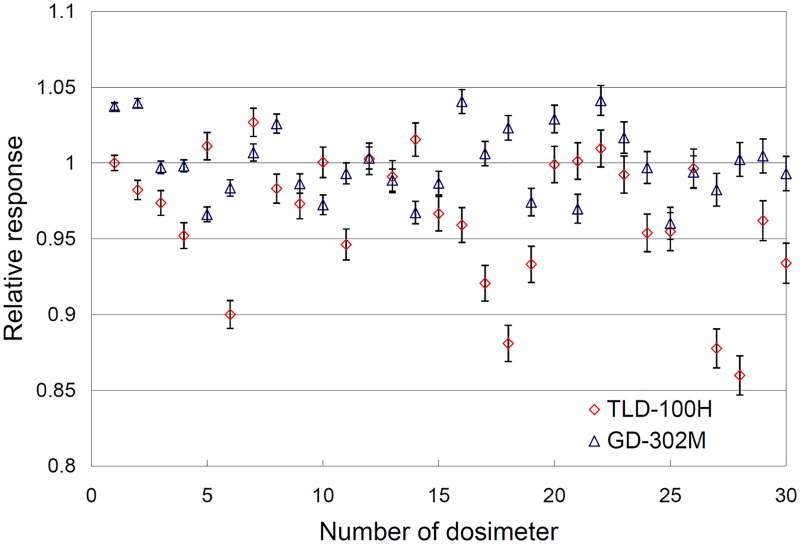
Reproducibility test for the TLD-100H and GD-302M readouts. The relative response represents the variation among the individual dosimeters, and is calculated by dividing the individual reading by the average of the 30 readings. The error bars show the variations in the reading reproducibility.

**Fig 4:**
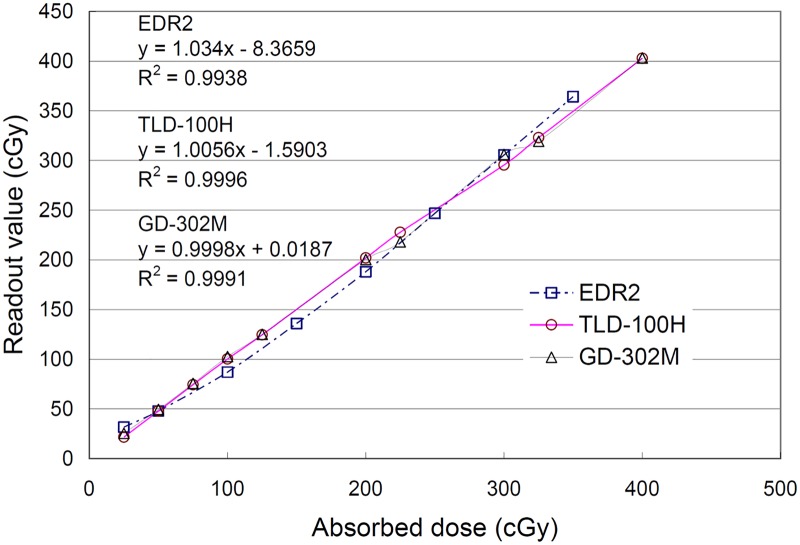
Linearity response for the EDR2 film, TLD-100H and GD-302M. The coefficients of variation for each dosimeter were less than 3.

Readout reproducibility was evaluated after multiple irradiations. A reproducibility analysis was conducted with the TLD-100H and GD-302M. The CVs analyzed for the TLD-100H were between 0.99% and 3.00%, whereas they were between 0.48% and 2.98% for the GD-302M. The results are shown in Fig 3. In this study, the CV of each dosimeter (TLD-100H, GD-302M and EDR2) was less than 3. Dose linearity was evaluated with the TLD-100H, GD-302M, and EDR2 film from 25 to 400 cGy. A regression curve and coefficient of determination (R-squared) were obtained for the readout dose and irradiated dose. As the coefficient approached 1, the relationship between the readout dose and irradiated dose was linear. The R-squared value for the TLD-100H, GD-302M and EDR2 film were 0.9996, 0.9991, and 0.9938, respectively, as shown in Fig 4.

### Lateral dose profile measurement

**Fig 5:**
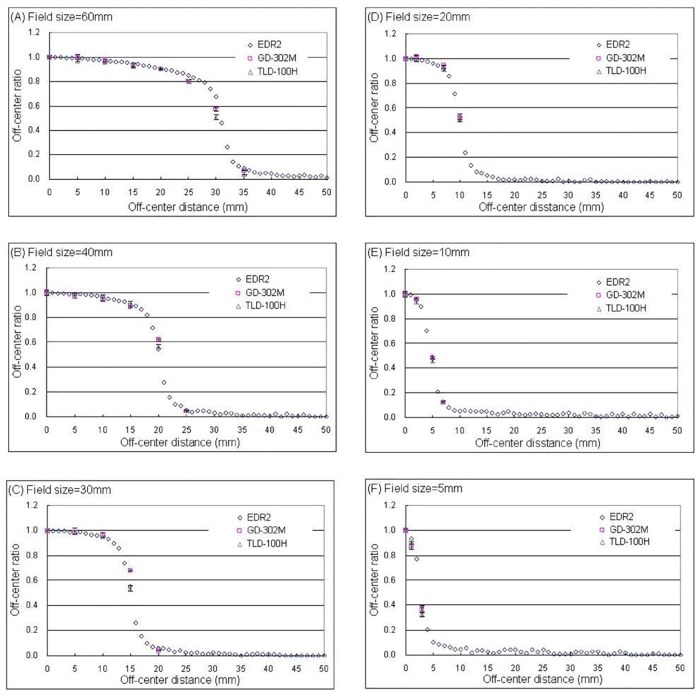
Results for the CyberKnife lateral dose profile measurement. (A), (B), (C), (D), (E), and (F) denote the circular collimator sizes 60, 40, 30, 20, 10, and 5 mm, respectively.

The lateral dose profile measurement results for the GD-302M, TLD-100H, and EDR2 film are shown in Fig 5. For circular collimator sizes between 60 to 20 mm, the measurement discrepancies for the GD-302M, TLD-100H, and EDR2 were less than 3%. For circular collimator sizes less than 10 mm, there was no uniform dose due to lateral electron disequilibrium. The lateral dose profile showed steep changes, which resulted in measurement discrepancies greater than 3% among the different dosimeters.

### Percent depth dose measurement

**Fig 6:**
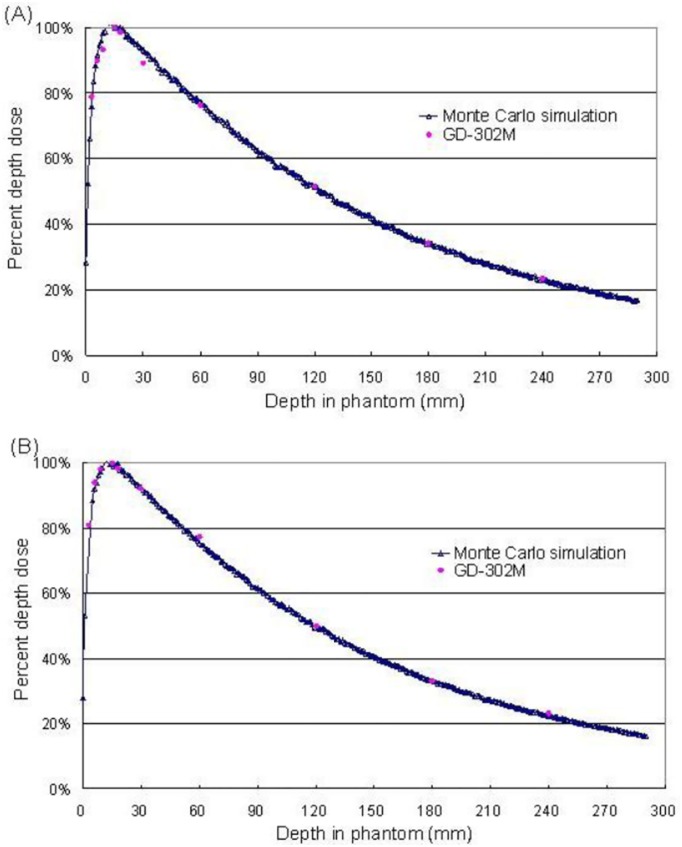
Comparison of the percent depth dose curve between the actual measurements and the Monte Carlo simulation for circular collimators of sizes (A) 60 mm and (B) 40 mm.

The comparison of the percent depth dose between the GD-302M measurements and the Monte Carlo simulation for CyberKnife circular collimators of 40 and 60 mm are shown in Fig 6. Twelve points were selected in this study for the dose measurements, and the CV of each point was less than 3%.

### Monte Carlo simulation

A comparison of the OMEGA/BEAM simulation and the GD-302M measured results is shown in Fig 6(A) for a 60 mm circular collimator. As indicated, the discrepancy in the build-up region was 4.00%, 5.23% and less than 1.84% at distances of 3 mm, 9 mm, or greater than 9 mm, respectively. The build-up region discrepancy was mainly affected by the electron disequilibrium, which led to greater measurement inaccuracy. When the circular collimator size was 40 mm, the discrepancy between the OMEGA/BEAM simulation and GD-302M measured results in the build-up region was less than 3.32%, and the discrepancy beyond the build-up region was less than 2.64% (Fig 6(B)).

**Fig 7:**
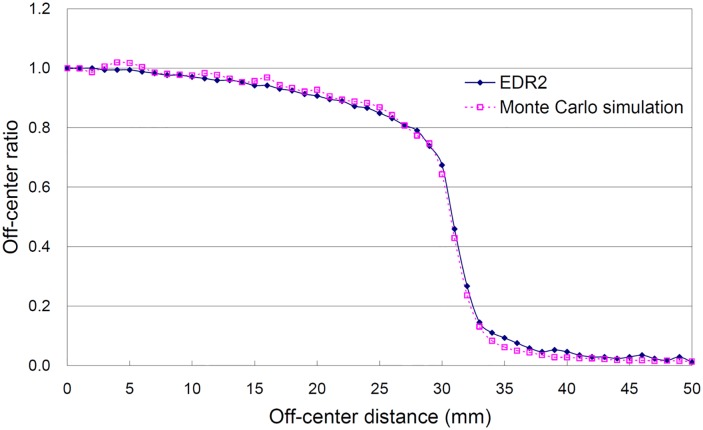
Comparison of the lateral curve for the actual measurements and the Monte Carlo simulation. The circular collimator size was 60 mm.

A comparison of the lateral dose profiles is shown in Fig 7 for the OMEGA/BEAM simulation and the EDR2 film. When distance was less than 29 mm, the measurement discrepancy of the EDR2 film relative to the OMEGA/BEAM simulation was less than 2.76%. The discrepancy was greater than 2% for points at 4, 5, 16, 20, 25 and 28 mm away from the center and less than 1.87% for the remaining points that were more than 30 mm away from the center. When the distances were 30 to 33 mm away from the center, the calculation from the OMEGA/BEAM simulation was underestimated by 4.86% to 13.27% relative to the EDR2 film. When the distance from the center was greater than 34 mm, the discrepancies between the OMEGA/BEAM and the EDR2 measurements were apparent without the flattening filter. Not enough photons reached the collimator edge, which led to inaccuracies in the OMEGA/BEAM calculations of 2.50% to 6.60%.

### Anthropomorphic phantom dose measurement

The calculated values from the CyberKnife treatment planning system and dose measurements in an anthropomorphic phantom with the GD-302M are compared in Table 1. The GD-302M measured value was an average of the measurements at the same location, whereas the calculated value from the CyberKnife planning system was an average of 5 dose calculation points inside the effective readout area for the GD-302M. The dose measurement for the GD-302M was 2840.63 cGy (CV 2.93%), and the average dose from the CyberKnife planning system was 2987.22 cGy (CV 0.08%). There is a 4.91% difference between the GD-302M measured value and the CyberKnife treatment planning system’s calculated value.

**Table 1:** Measurement results for Cyber Knife treatment planning system (TPS) and GD-302M dosimeter.

	Dose (cGy)	C.V.
GD-302M	2840.63 ± 83.20	2.93%
TPS	2987.22 ± 2.31	0.08%

## Conclusions

The GD-302M, TLD-100H, and EDR2 film could accurately evaluate the relative output factor for circular collimator sizes larger than 10 mm. When the circular collimator size was less than 7.5 mm, the EDR2 film exhibited better spatial resolution and is recommended for the dose measurements. The percent depth dose simulation calculation and measured value exhibited a discrepancy of 5.23% in the build-up region and less than 2.64% discrepancy in other regions. The lateral dose profile simulation and measured value in the uniform dose area exhibited a discrepancy of less than 2.76%. According to the results of the anthropomorphic phantom experiment, RPLGD is a satisfactory measurement tool for the dose verification of the CyberKnife treatment.

## Supporting Information

S1 File The original data of Fig 3 in this study. (PDF)

S2 File The original data of Fig 4 in this study. (PDF)

S3 File The original data of Fig 5 in this study. (PDF)

S4 File The original data of Fig 6 in this study. (PDF)

S5 File The original data of Fig 7 in this study. (PDF)
