# Supplementary material for: Feasibility Study on Applying Radiophotoluminescent Glass Dosimeters for CyberKnife SRS Dose Verification
Source: PLoS One. 2017 Jan 3;12(1):e0169252. doi: 10.1371/journal.pone.0169252 (PMC5207685; doi:10.1371/journal.pone.0169252)
Supplement: S1 File — (PDF) [file pone.0169252.s001.pdf]

Fig. 3. Reproducibility test for the TLD-100H and GD-302M readouts.

	TLD	GD
1	1	1.037343
2	0.982185	1.039449
3	0.973604	0.997108
4	0.952006	0.998106
5	1.011094	0.966073
6	0.900018	0.983475
7	1.026815	1.006862
8	0.983082	1.025926
9	0.973076	0.986357
10	1.000425	0.972391
11	0.946224	0.993118
12	1.002779	1.003204
13	0.990935	0.988684
14	1.015469	0.967292
15	0.966501	0.986689
16	0.959009	1.040557
17	0.920651	1.006197
18	0.880924	1.023155
19	0.933162	0.974164
20	0.999	1.02903
21	1.001268	0.969731
22	1.009546	1.041222
23	0.992303	1.016727
24	0.953807	0.996997
25	0.954694	0.960199
26	0.996306	0.994005
27	0.877618	0.982367
28	0.859778	1.002318
29	0.961915	1.004645
30	0.933846	0.993007
