# Supplementary material for: Feasibility Study on Applying Radiophotoluminescent Glass Dosimeters for CyberKnife SRS Dose Verification
Source: PLoS One. 2017 Jan 3;12(1):e0169252. doi: 10.1371/journal.pone.0169252 (PMC5207685; doi:10.1371/journal.pone.0169252)
Supplement: S2 File — (PDF) [file pone.0169252.s002.pdf]

Fig. 4. Linearity response for the EDR2 film, TLD-100H and GD-302M.

	EDR2	TLD-100H	GD-302M
25	31.52	21.58	25.45
50	47.83	47.79	49.45
75		74.31	75.67
100	86.96	100.29	102.62
125		124.57	125.00
150	135.87		
200	188.04	201.89	200.33
225		227.73	218.01
250	246.74		
300	305.43	295.47	305.99
325		322.91	319.23
350	364.13		
400		402.77	403.06
