# Supplementary material for: Feasibility Study on Applying Radiophotoluminescent Glass Dosimeters for CyberKnife SRS Dose Verification
Source: PLoS One. 2017 Jan 3;12(1):e0169252. doi: 10.1371/journal.pone.0169252 (PMC5207685; doi:10.1371/journal.pone.0169252)
Supplement: S3 File — (PDF) [file pone.0169252.s003.pdf]

Fig. 5. Results for the CyberKnife lateral dose profile measurement.

	60mm	40mm	30mm	20mm	10mm	5mm
OCD	EDR2	EDR2	EDR2	EDR2	EDR2	EDR2
0	1.000	1.000	1.000	1.000	1.000	1.000
1	1.000	1.000	1.000	1.000	0.993	0.935
2	1.000	0.994	1.000	0.994	0.958	0.769
3	0.994	0.994	1.000	0.987	0.894	0.389
4	0.994	0.988	0.988	0.975	0.704	0.204
5	0.994	0.988	0.988	0.962	0.493	0.102
6	0.988	0.988	0.988	0.943	0.211	0.083
7	0.983	0.982	0.976	0.912	0.127	0.074
8	0.977	0.976	0.964	0.856	0.077	0.065
9	0.977	0.970	0.958	0.713	0.056	0.046
10	0.971	0.964	0.946	0.506	0.050	0.046
11	0.965	0.953	0.927	0.238	0.056	0.056
12	0.959	0.947	0.897	0.138	0.050	0.018
13	0.959	0.934	0.855	0.087	0.050	0.037
14	0.953	0.929	0.739	0.075	0.050	0.037
15	0.942	0.911	0.551	0.056	0.050	0.028
16	0.942	0.893	0.267	0.044	0.042	0.018
17	0.930	0.863	0.157	0.031	0.028	0.028
18	0.924	0.822	0.103	0.025	0.042	0.046
19	0.912	0.714	0.073	0.025	0.050	0.046
20	0.907	0.548	0.061	0.025	0.035	0.046
21	0.895	0.280	0.061	0.019	0.028	0.028
22	0.890	0.161	0.048	0.031	0.021	0.018
23	0.872	0.101	0.036	0.031	0.028	0.046
24	0.866	0.089	0.043	0.025	0.035	0.028
25	0.849	0.060	0.030	0.012	0.028	0.046
26	0.831	0.042	0.024	0.012	0.021	0.028
27	0.808	0.054	0.030	0.012	0.021	0.010
28	0.790	0.054	0.018	0.031	0.021	0.010
29	0.738	0.048	0.018	0.006	0.035	0.010
30	0.674	0.036	0.030	0.012	0.042	0.010
31	0.459	0.024	0.024	0.006	0.021	0.028
32	0.267	0.036	0.018	0.000	0.014	0.028
33	0.145	0.030	0.018	0.019	0.035	0.018
34	0.111	0.018	0.012	0.025	0.028	0.028
35	0.093	0.012	0.000	0.000	0.014	0.028
36	0.076	0.012	0.012	0.006	0.007	0.010
37	0.058	0.018	0.012	0.006	0.014	0.000
38	0.046	0.012	0.018	0.000	0.028	0.010
39	0.052	0.012	0.012	0.006	0.014	0.028
40	0.046	0.006	0.012	0.000	0.028	0.018
41	0.035	0.024	0.012	0.000	0.007	0.000
42	0.029	0.000	0.006	0.000	0.028	0.000
43	0.029	0.024	0.000	0.000	0.014	0.000
44	0.023	0.006	0.000	0.000	0.014	0.000
45	0.029	0.006	0.000	0.006	0.028	0.010
46	0.035	0.018	0.000	0.000	0.007	0.010
47	0.023	0.006	0.000	0.000	0.021	0.000
48	0.017	0.000	0.000	0.006	0.000	0.000
49	0.029	0.000	0.000	0.000	0.007	0.000

60								
OCD	0	5	10	15	20	25	30	35
GD	1.000	1.001	0.971	0.929	0.901	0.800	0.569	0.071
TLD	1.000	0.970	0.959	0.937	0.905	0.801	0.506	0.039

40						
OCD	0	5	10	15	20	25
GD	1.000	0.968	0.948	0.889	0.623	0.045
TLD	1.000	0.977	0.964	0.915	0.564	0.049

30					
OCD	0	5	10	15	20
GD	1.000	0.986	0.970	0.682	0.047
TLD	1.000	0.996	0.952	0.536	0.042

20				
OCD	0	2	7	10
GD	1.000	1.017	0.947	0.519
TLD	1.000	1.000	0.911	0.516

10				
OCD	0	2	5	7
GD	1.000	0.961	0.482	0.119
TLD	1.000	0.938	0.456	0.131

5			
OCD	0	1	3
GD	1.000	0.871	0.365
TLD	1.000	0.899	0.326
