# Supplementary material for: Feasibility Study on Applying Radiophotoluminescent Glass Dosimeters for CyberKnife SRS Dose Verification
Source: PLoS One. 2017 Jan 3;12(1):e0169252. doi: 10.1371/journal.pone.0169252 (PMC5207685; doi:10.1371/journal.pone.0169252)
Supplement: S4 File — (PDF) [file pone.0169252.s004.pdf]

Fig. 6. Comparison of the percent depth dose curve between the actual measurements and the Monte Carlo simulation for circular collimators of sizes (60 m

depth	PDD	depth	GD
0	0.283	3	0.790
1	0.523	6	0.899
2	0.662	9	0.932
3	0.759	12	1.007
4	0.834	15	1.000
5	0.884	18	0.986
6	0.916	30	0.892
7	0.946	60	0.762
8	0.959	120	0.516
9	0.983	180	0.343
10	0.988	240	0.234
11	0.992		
12	1.006		
13	1.015		
14	1.000		
15	1.000		
16	0.997		
17	0.999		
18	0.998		
19	0.996		
20	0.990		
21	0.971		
22	0.976		
23	0.966		
24	0.961		
25	0.957		
26	0.948		
27	0.949		
28	0.940		
29	0.936		
30	0.932		
31	0.925		
32	0.923		
33	0.915		
34	0.905		
35	0.902		
36	0.902		
37	0.893		
38	0.877		
39	0.872		
40	0.863		
41	0.868		
42	0.863		
43	0.858		
44	0.851		
45	0.839		
46	0.843		
47	0.840		
48	0.836		
49	0.828		
50	0.814		
51	0.815		
52	0.808		
53	0.804		
54	0.796		
55	0.792		
56	0.786		
57	0.780		
58	0.784		
59	0.775		
60	0.775		
61	0.762		
62	0.763		
63	0.757		
64	0.748		

65	0.743
66	0.737
67	0.731
68	0.727
69	0.720
70	0.719
71	0.718
72	0.712
73	0.704
74	0.712
75	0.697
76	0.689
77	0.686
78	0.680
79	0.671
80	0.676
81	0.665
82	0.661
83	0.652
84	0.655
85	0.650
86	0.641
87	0.641
88	0.635
89	0.628
90	0.624
91	0.613
92	0.618
93	0.614
94	0.611
95	0.605
96	0.600
97	0.600
98	0.593
99	0.579
100	0.575
101	0.573
102	0.579
103	0.574
104	0.572
105	0.570
106	0.561
107	0.558
108	0.558
109	0.556
110	0.551
111	0.546
112	0.545
113	0.539
114	0.528
115	0.526
116	0.519
117	0.523
118	0.521
119	0.520
120	0.514
121	0.513
122	0.501
123	0.500
124	0.496
125	0.497
126	0.493
127	0.485
128	0.487
129	0.486
130	0.476
131	0.468
132	0.471

133	0.464
134	0.463
135	0.462
136	0.461
137	0.458
138	0.461
139	0.449
140	0.450
141	0.444
142	0.442
143	0.439
144	0.435
145	0.433
146	0.427
147	0.429
148	0.427
149	0.430
150	0.421
151	0.417
152	0.412
153	0.405
154	0.404
155	0.404
156	0.402
157	0.393
158	0.397
159	0.394
160	0.395
161	0.391
162	0.389
163	0.381
164	0.379
165	0.378
166	0.376
167	0.372
168	0.369
169	0.366
170	0.363
171	0.363
172	0.356
173	0.354
174	0.355
175	0.350
176	0.351
177	0.350
178	0.348
179	0.341
180	0.338
181	0.342
182	0.340
183	0.334
184	0.333
185	0.331
186	0.331
187	0.329
188	0.327
189	0.325
190	0.316
191	0.318
192	0.318
193	0.320
194	0.316
195	0.311
196	0.308
197	0.309
198	0.306
199	0.303
200	0.298

201	0.294
202	0.295
203	0.292
204	0.288
205	0.288
206	0.288
207	0.286
208	0.286
209	0.284
210	0.279
211	0.281
212	0.279
213	0.276
214	0.272
215	0.270
216	0.271
217	0.271
218	0.267
219	0.267
220	0.266
221	0.263
222	0.258
223	0.262
224	0.259
225	0.252
226	0.255
227	0.252
228	0.249
229	0.247
230	0.247
231	0.244
232	0.245
233	0.242
234	0.239
235	0.240
236	0.243
237	0.234
238	0.231
239	0.231
240	0.230
241	0.230
242	0.230
243	0.224
244	0.223
245	0.225
246	0.223
247	0.219
248	0.221
249	0.215
250	0.215
251	0.210
252	0.215
253	0.212
254	0.213
255	0.211
256	0.208
257	0.206
258	0.211
259	0.206
260	0.203
261	0.202
262	0.204
263	0.203
264	0.199
265	0.196
266	0.198
267	0.195
268	0.195

269	0.189
270	0.191
271	0.189
272	0.188
273	0.184
274	0.181
275	0.185
276	0.185
277	0.182
278	0.181
279	0.180
280	0.175
281	0.175
282	0.175
283	0.176
284	0.175
285	0.174
286	0.171
287	0.167
288	0.166
289	0.168
290	0.167
