# Supplementary material for: Feasibility Study on Applying Radiophotoluminescent Glass Dosimeters for CyberKnife SRS Dose Verification
Source: PLoS One. 2017 Jan 3;12(1):e0169252. doi: 10.1371/journal.pone.0169252 (PMC5207685; doi:10.1371/journal.pone.0169252)
Supplement: S5 File — (PDF) [file pone.0169252.s005.pdf]

Fig. 7. Comparison of the lateral curve for the actual measurements and the Monte Carlo simulation. The circular collimator size was 60 mı

OCR	EDR2	MC
0	1	1
1	1	0.998974
2	1	0.986325
3	0.9941	1.00547
4	0.9941	1.018803
5	0.9941	1.017094
6	0.9881	1.003419
7	0.9826	0.984957
8	0.9766	0.980513
9	0.9766	0.977778
10	0.9707	0.975043
11	0.9651	0.982906
12	0.9592	0.977436
13	0.9592	0.964444
14	0.9532	0.952821
15	0.9418	0.956239
16	0.9418	0.968547
17	0.9303	0.942564
18	0.9243	0.933333
19	0.9124	0.921026
20	0.9069	0.927179
21	0.895	0.904957
22	0.8895	0.893675
23	0.872	0.887179
24	0.8661	0.882393
25	0.8486	0.867692
26	0.8312	0.842051
27	0.8078	0.807179
28	0.7904	0.773333
29	0.7381	0.746667
30	0.6743	0.643077
31	0.4592	0.428718
32	0.2674	0.236068
33	0.1454	0.131043
34	0.1105	0.083214
35	0.0931	0.061641
36	0.0757	0.049915
37	0.0583	0.044103
38	0.0463	0.035316
39	0.0523	0.028222
40	0.0463	0.027573
41	0.0349	0.024694
42	0.0289	0.024106
43	0.0289	0.021562
44	0.0234	0.018851
45	0.0289	0.017231
46	0.0349	0.017282
47	0.0234	0.015668
48	0.0174	0.016578
49	0.0289	0.015617
50	0.0115	0.013241
